# Groups: knowledge spreadsheets for symbolic biocomputing

**DOI:** 10.1093/database/bat061

**Published:** 2013-09-14

**Authors:** Michael Travers, Suzanne M. Paley, Jeff Shrager, Timothy A. Holland, Peter D. Karp

**Affiliations:** Bioinformatics Research Group, SRI International, Menlo Park, CA 94025, USA

## Abstract

Knowledge spreadsheets (KSs) are a visual tool for interactive data analysis and exploration. They differ from traditional spreadsheets in that rather than being oriented toward numeric data, they work with symbolic knowledge representation structures and provide operations that take into account the semantics of the application domain. ‘Groups’ is an implementation of KSs within the Pathway Tools system. Groups allows Pathway Tools users to define a group of objects (e.g. groups of genes or metabolites) from a Pathway/Genome Database. Groups can be transformed (e.g. by transforming a metabolite group to the group of pathways in which those metabolites are substrates); combined through set operations; analysed (e.g. through enrichment analysis); and visualized (e.g. by painting onto a metabolic map diagram). Users of the Pathway Tools-based BioCyc.org website have made extensive use of Groups, and an informal survey of Groups users suggests that Groups has achieved the goal of allowing biologists themselves to perform some data manipulations that previously would have required the assistance of a programmer.

**Database URL**: BioCyc.org.

## Introduction

A long-standing problem in computing is that of providing non-programmers with intuitive, yet powerful tools for manipulating and analysing sets of entities. For example, a number of bioinformatics database websites provide users with powerful tools for composing database queries, but once a user obtains the query results, they are largely on their own. What if a user wants to store the query results for future reference, or combine them with other query results, or transform the results, or share them with a colleague? Sets of entities of interest arise in other contexts for life scientists, such as the entities that are identified as significantly perturbed in a high-throughput experiment (e.g. a set of differentially occurring metabolites), or a set of genes of interest that emerge from an experimental investigation.

We observe that spreadsheets have become a dominant form of end-user programming and data analysis for scientists. Although traditional spreadsheets provide a compelling interaction model, and are excellent tools for the manipulation of the tables of numbers that are typical of accounting and data analysis problems, they are less easily used with the complex symbolic computations typical of symbolic biocomputing. For example, they cannot perform semantic transformations such as converting a gene list to the list of pathways the genes act in.

We coined the term knowledge spreadsheet (KS) to describe spreadsheets that are characterized by their ability to manipulate semantic objects and relationships instead of just numbers and strings. Both traditional spreadsheets and KSs represent data in tabular structures, but in a KS the contents of a cell will typically be an object from a knowledge base (KB) [such as a MetaCyc (1) frame or a URI entity from an RDF store]. Given that a column in a KS will typically contain objects of the same ontological type, a KS can offer high-level semantically knowledgeable operations on the data. For example, given a group with a column of metabolites, a semantic operation could create a parallel column in which each cell contained the reactions that produced that metabolite. Another difference between our implementation of KSs and traditional spreadsheets is that cells in our KSs can contain multiple values.

The KS system described in this article works with frame objects in a KB and offers a variety of operations for semantically transforming such objects; analyzing them; importing, exporting and displaying them; saving them persistently; and sharing them with colleagues. We call the implementation of KSs within the Pathway Tools (2) software system ‘Groups’. Pathway Tools has a web server mode that underlies the BioCyc.org website and other websites listed at (3). Online documentation for Groups can be found at (4). To experiment with Groups at BioCyc, go to BioCyc.org, create an account (groups are stored in conjunction with user accounts), and click Groups under the Tools menu. Note the appearance of an additional Groups menu item.

A generic KS can traverse explicit relationships between objects (such as those defined by semantic web standards like RDF); a domain-specific KS will also provide built-in operators for specific classes of objects. The Groups implementation offers both types of operations. The ‘add property column’ menu is populated by examination of the underlying KB and presents the raw relationships that it encodes; it would work equally well if the underlying KB was switched to a different domain (e.g., automobile parts). The ‘add transform’ menu works in a similar fashion but offers a domain-specific set of transformations that may involve semantic computation.

Because traditional spreadsheets have no representation of the semantics of the data being manipulated, it is entirely up to the user to make sure the operations make sense. Given the use of spreadsheets by non-professional programmers, the error rate is high. A recent well-publicized study (5) showed that an earlier influential analysis of economic data was seriously flawed, in part due to Excel programming errors, and entire professional groups and conferences (6) are devoted to analysing and recovering from similar disasters (5, 6). Although a KS obviously cannot eliminate all computational errors, because users interact at a higher level, we posit that opportunities for users to make these sorts of syntactical/mechanical errors will be reduced.

Groups simplify both the software engineering and the human–computer interaction of Pathway Tools because Groups ties together several existing functionalities of Pathway Tools, such as tools for generating sets of objects, analysing sets of objects and displaying sets of objects. Implementing ad-hoc connections among all such modules would require O(n^2^) separate software efforts, such as connecting query result management with enrichment analysis and with painting onto regulatory and metabolic maps. Instead, we are connecting these modules via Groups at a cost of O(n). To serve such a function, Groups must be designed in a general and abstract way.

### Relation to previous work

Web Groups is influenced by a number earlier strands of research, including efforts to build end-user programming tools for scientific applications, and software to generalize spreadsheets to hold complex data and explore semantic relationships.

#### Visual data flow models

The most common approach to end-user programming for scientific applications involves visual data flow models, where computations are defined by interactively wiring together components. Examples include the commercial systems Pipeline Pilot and LabView, and the academic/open source systems Taverna (7) and Knime (8). Although these systems are powerful, they tend not to scale well in complexity (9). Unlike spreadsheets, the visual interface emphasizes the program rather than data.

Galaxy is another web-based system for end-user creation of scientific computational workflows (10). It is closer to Web Groups in spirit because it emphasizes manipulations on tabular data.

#### Spreadsheets

Spreadsheets are of course a widely known technique for end-user programmable manipulation of data. Semantic spreadsheets, that is, spreadsheets that contain and compute over complex symbolic structures, are a more novel concept, but there are some predecessors. The most direct ancestor of Web Groups was a tabular knowledge manipulation tool prototyped in BioBike (11). Many other semantic web or other data systems offer tabular views that share some subset of web groups features; we mention only a few here.

Google Squared was a research project that presented the user with a tabular view of semantically related entities and their properties (12). As in Web Groups, entities were in rows, whereas columns expressed semantically derived relationships. Entities automatically extracted from Googles very large text corpus; the tool was chiefly intended for comparing sets of related entities, rather than for semantic computation, and it did not allow creation of derived groups.

Freebase.com (now also owned by Google) is a general-purpose semantic store, whose web UI has some semantic-spreadsheet features. A tabular view features rows of entities and columns express semantically related properties, which may be other frame objects in the KB. Filtering and sorting can be performed on these properties, but it is not possible to turn a column into a new group or display or derive columns transitively.

RightField adds biological ontology annotation capabilities to Excel spreadsheets. However, it is strictly for terminological semantics and does not provide any relationship or computational facilities(13).

## The Structure of Groups

Like a traditional spreadsheet, a KS consists of a set of cells organized into rows and columns. Cells contain data values such as numbers and strings. We refer to a KS as a group because typically each row in a knowledge spreadsheet describes one object from a KB; therefore, the set of rows in a knowledge spreadsheet corresponds to a group of KB objects.

**Figure 1.**
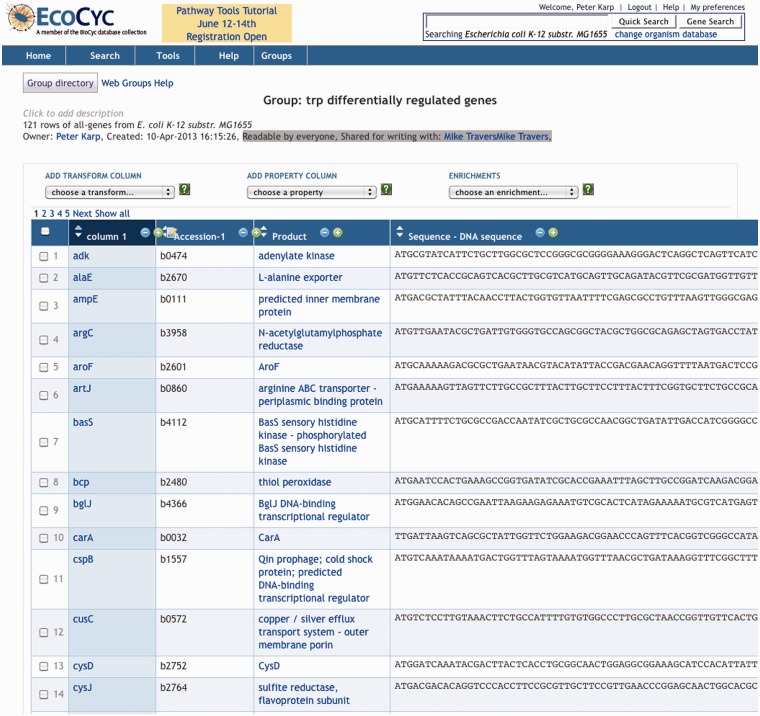
A group of genes (partial listing). The columns present are gene name, accession number, gene product and sequence (truncated).

**Figure 2.**
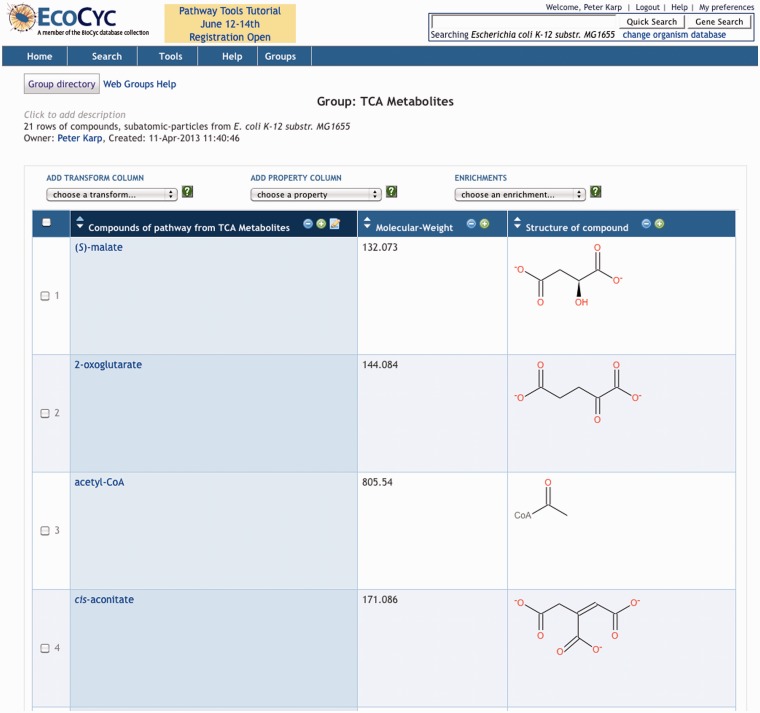
A group of compounds (partial listing).

For example, Figure 1 shows a group of *Escherichia coli* genes from the EcoCyc DB; each row corresponds to one gene. Figure 2 shows a group of metabolites. The user can determine which object properties are displayed as columns as shown in Figures 1 and 2; some columns are computed dynamically, such as the chemical structure diagram. Although object attributes and relationships are a common type of column in Groups, as we shall see shortly, they are not the only type of column.

The space of cell value types includes numbers, strings, KB objects, regions on nucleotide sequences and images (e.g. chemical structures).

All groups that users create are saved persistently in a MySQL database; each group is associated with the user’s BioCyc.org account.

### User interfaces for groups

Pathway Tools has two user interfaces for Groups, in its desktop mode and its web server mode; the majority of this article describes the latter, and all screen shots are from the web mode. Both interfaces support operations such as creating groups in a variety of ways, the addition and deletion of rows and columns, interactive editing of cells and of the group definition, etc.

The URL associated with a group is stable and can be bookmarked.

### Directories, metagroups, special groups, sharing of groups

Meta-groups. The Groups directory pages are implemented using meta-groups, which are groups whose objects are themselves groups, along with their derived properties. These groups have mechanisms that allow them to use SQL tables directly as their persistent store. This effectively allows the Groups’ directory pages to be generated using efficient SQL queries.

Special groups. Special groups are those whose contents are defined computationally rather than stored explicitly. Web Groups offers a set of prebuilt special groups based on the Pathway Tools ontology. For instance, a user who has set their current organism to *Bacillus subtilis* can navigate to special groups consisting of all the genes, or all metabolic reactions of that organism. Special groups cannot be edited, but the system makes it easy for a user to create an editable copy of a special group.

Sharing groups. By default, newly created groups are the private information of the user who created them. However, users may change the sharing status of a group to give read or write access to the group to specific other BioCyc users, or to all users. Thus, Groups can be used as a mechanism to enable data sharing and collaboration. Furthermore, we plan to implement a ‘publish’ sharing option for Groups that makes a group readable by the public, but locks the contents of the group from modification even by the group owner to as to create an archival record of the group. This mechanism will allow a group to be used in conjunction with a scientific publication, such as to make a gene set available for readers to analyse and manipulate (e.g. by transformation to create a new group).

## Generating, Importing and Exporting Groups

Several operations exist for creating and adding information to Groups, and for extracting information from Groups.

Groups can be created by importing data from a tab-delimited file. The first column of the file contains identifiers, common names or synonyms of objects in the KB with which the Group is associated. Additional columns, if present, can contain arbitrary data fields that are stored within the group; for example, a user might include with a gene group columns of gene-expression measurements.

Groups can also be created to hold the results of a web query, such as queries using the Structured Advanced Query Form (14). Furthermore, when the user is viewing an object page in a Pathway Tools website (such as a metabolite page), that object can be added to a Group.

The contents of a group can be exported to tab-delimited files.

## Operations on Groups

What most distinguishes KSs from traditional spreadsheets are the symbolic operations discussed in this section.

### Transformations on groups

A Groups transformation computes a new column for a group. Often that column is derived by following a database relationship from each group member, but in some cases the column is derived from a combination of multiple database relationships plus computation that yields some meaningful biological relationship (e.g. the multiple queries needed to transform a gene set into the set of metabolic pathways in which the gene products catalyse reactions).

**Figure 3.**
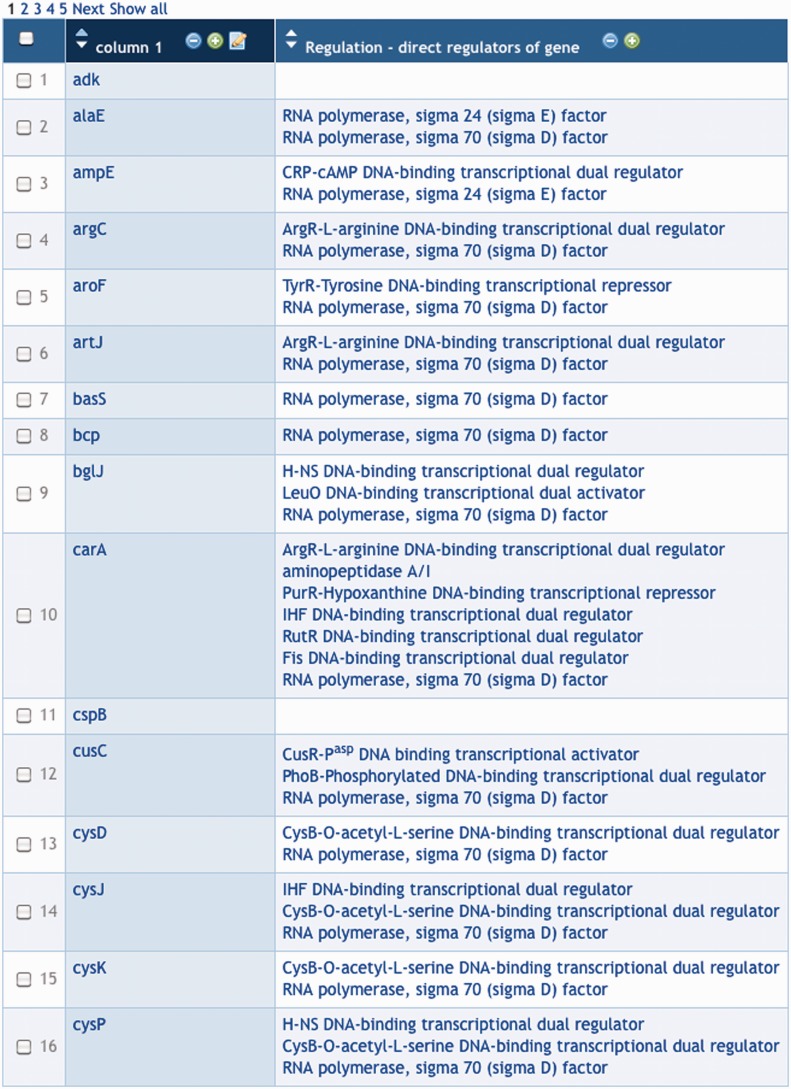
Column 2 of this group was produced through a transform that computes the regulators (transcription factors and sigma factors) of each gene in column 1.

For example, given the group of genes in Figure 1, we can apply a transformation to that group that computes the transcriptional regulator(s) of each gene in the group as a new column value for that gene—see Figure 3.

**Figure 4.**
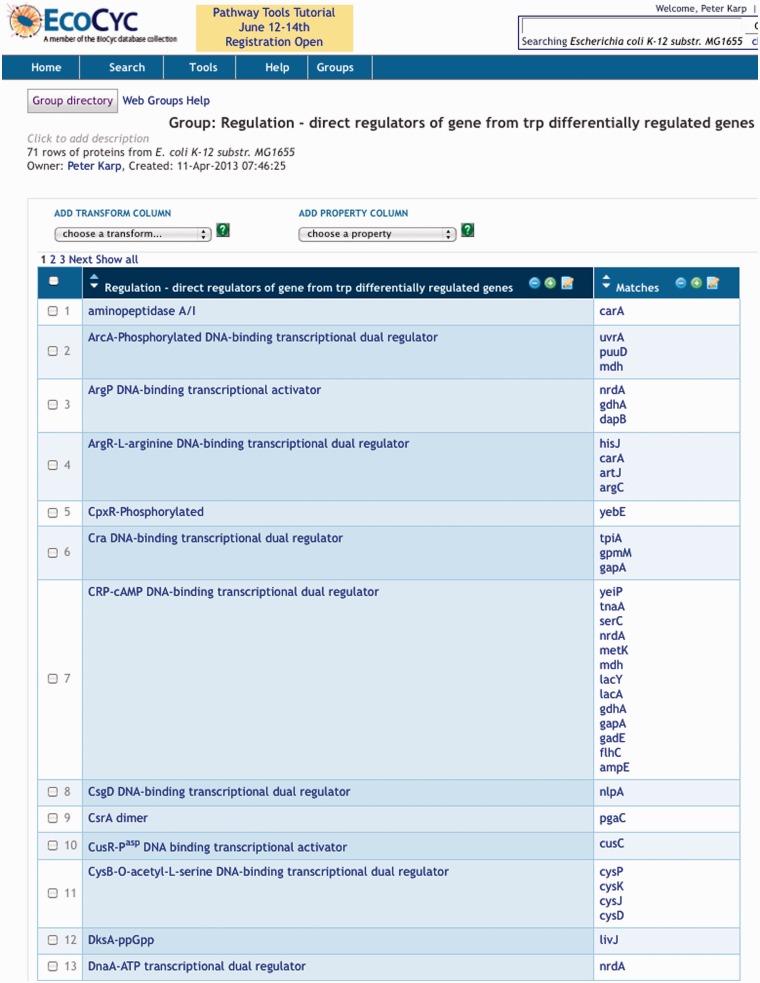
Column 1 of this group consists of the union of all regulators found in column 2 of Figure 3. Column 2 of this group shows for each regulator, the genes from column 1 of Figure 3 controlled by the regulator.

Often the user wants to create a new group consisting of the non-redundant set of all values in a column created by a transformation. In the preceding example, that new group consists of the set of all regulators of the original gene group, which can be created with a single mouse click; the results are shown in Figure 4. In this style of analysis, one can think of a transformation as taking one group as input (here, the starting gene group) and producing another group as output (here, the set of all regulators of those genes).

Another example transformation on genes is as follows. This transformation reduces the size of a gene group by computing the functional complexes encoded by that group, removing genes that are in a sense redundant if they encode subunits of the same complex. This transformation is useful for gene expression analysis because it compresses a gene set to a smaller gene product set, e.g. if a gene set contained the eight genes encoding subunits of the *E. coli* ATP synthase F1 complex, they would be replaced in the transformed group with the single ATP synthase complex. For a starting gene C, if the product P of C forms one or more protein complexes, the transformation returns those complexes; if no complexes are formed the transformation returns P.

Other transformations available for Groups of genes include the following. We can transform a gene group C to: (i) regulation-related sets, including the set of genes regulated by C, the set of genes that regulate genes in C, the set of regulatory binding sites upstream of the operon of C, the set of promoters of C, the set of operons containing C, genes in the same operon as C and regulatory diagrams depicting regulatory influences on genes in G; (ii) reactions catalysed by G; (iii) the set of metabolic pathways in which the products of G catalyse reactions; (iv) gene ontology terms annotated to G; and (v) comparative sets, including the orthologs of G in another PGDB; (vi) the set of transcription-factor binding sites upstream of the genes; (vii) the set of promoters regulated by the genes; (viii) the sequence region of the coding region of each gene.

Different sets of transformations are available for the different datatypes supported by Pathway Tools. A somewhat more elaborate use case for Groups is: Given a metabolic pathway, find all genes in that pathway, and then find all known transcription-factor binding sites upstream of those genes. A variation of the last step entails retrieving the nucleotide sequence 50 bases upstream and downstream of the promoter upstream of each gene. Groups can accomplish this use case.

Transformations on metabolic pathways. A group of metabolic pathways P can be transformed to (i) the set of reactions in P; (ii) the set of enzymes that catalyse reactions in P; (iii) the set of genes whose products catalyse reactions in P; or (iv) the set of metabolites that are produced, or consumed, or both, by reactions in P.

Transformations on chemical compounds. A group of chemical compounds C can be transformed to (i) the set of reactions that produce C, that consume C, or both; (ii) the metabolic pathways that produce C, that consume C, or both; (iii) the set of enzymes that are activated by C, or that are inhibited by C; (iv) the set of proteins that bind C as ligands; or (v) the set of genes that are regulated by C, such as if C is a transcription-factor ligand.

Transformations on promoters. A group of promoters P can be transformed to (i) binding sites that regulate P; (ii) genes downstream of (and controlled by) P; (iii) the sequence region of P.

Transformations on sequence regions. Groups can contain sequence regions, which consist of start/end pairs on a specified replicon. A sequence region R can be transformed to (i) The length of R; (ii) The gene nearest to R; (iii) The sequence of R; (iv) A new region derived from R, e.g. the user can specify that the new region be computed by subtracting 100 from the start of R and by adding 100 to the end of R.

Transformations are not available for all conceivable group columns. For example, a column consisting of a set of numbers or a set of strings has no applicable transformations.

### Group enrichments

Consider the analysis of a gene expression experiment in which 200 genes are found to be significantly up or down-regulated and are collected into a group. A biologist may want to know which set of biological processes (such as cell division) or biological pathways is most relevant to that group of 200 genes. Enrichment analysis is a statistical analysis tool that answers this type of question by determining the degree to which the set of genes known to be involved in a given biological process or pathway is statistically over-represented in the group of input genes, relative to chance. Because high-throughput experiments are noisy, and genes and compounds can participate in multiple biological processes or pathways, in the context of the above example it would be a mistake to assume that all the pathways in which at least one gene from a group of 200 genes is involved participate in the phenomenon studied in the gene expression experiment. Enrichment analysis enables users to statistically distinguish the pathways, thereby differentiating the phenomenon that underlies the expression experiment from the ones that contain genes from the group purely by happenstance.

Enrichment analysis was initially described (15–17) for lists of genes obtained using microarray experiments and for Gene Ontology (GO) terms. On a Pathway Tools web site, a Group of genes can be subjected to enrichment analysis with respect to the following categories.

- GO terms (each of the three GO ontologies, Molecular Function, Biological Process and Cellular Location, is considered a different enrichment problem).
- Pathways and classes of pathways.
- Transcriptional regulators, i.e. is there one or more transcription factors whose action could explain the co-incidence of the genes in the group?

The implementation provides three statistical tests: Fisher Exact, Fisher Exact Parent–Child Union and Fisher Exact Parent–Child Intersection. Three options for multiple-testing correction are provided: Bonferroni correction, Benjamini–Hochberg correction and Benjamini–Yekutieli correction.

In addition, there is an option to perform a single enrichment analysis that combines all of these factors: all three GO ontologies, pathways and transcriptional regulators.

A group of metabolites, such as those from a metabolomics experiment, can be subjected to enrichment analysis with respect to the set of pathways and classes of pathways that include those metabolites as substrates.

**Figure 5.**
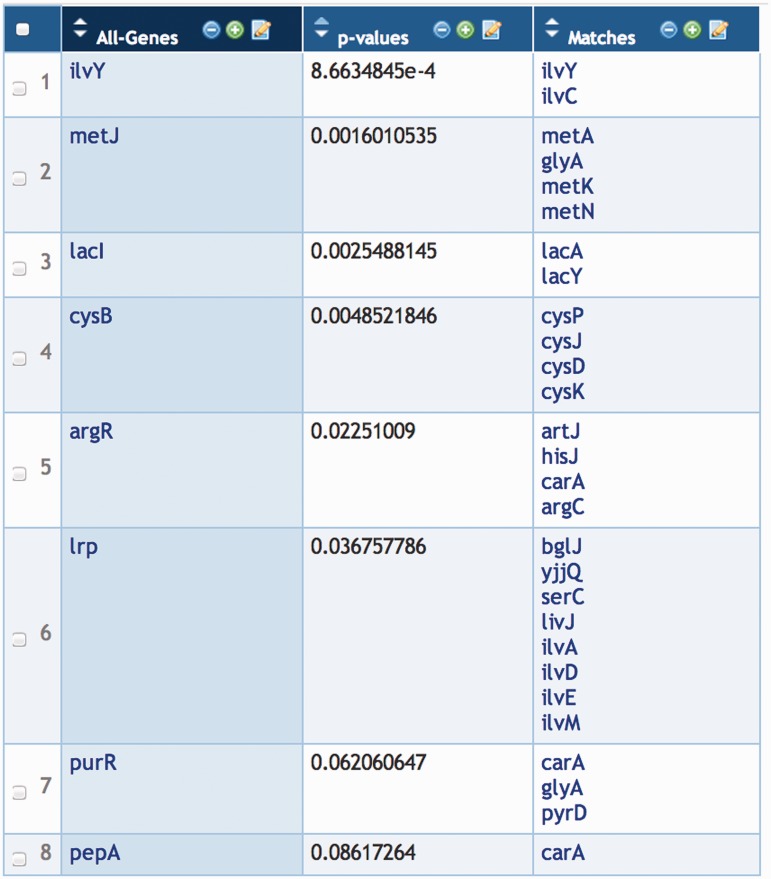
A group of *E. coli* transcription factor genes generated as the result of an enrichment analysis. The genes in the third column are those members of the initial gene group that are regulated by the corresponding transcription factor in the first column.

The output of an enrichment analysis is a new group of objects (GO terms, pathways or transcription factor genes, depending on the type of enrichment analysis) whose *P*-value (the probability that the genes or metabolites associated with that object would appear in the original group purely by chance) is less than some user-specified threshold. An example, in which a group of genes from a differential expression experiment was analysed with respect to transcriptional regulators, is shown in Figure 5.

### Set and filter operations

A full complement of set operations is provided for Groups, such as creating a new group that contains the union, intersection and set difference of two groups.

Filter operations allow the user to select objects from a group (to update the group or form a new group) by enlisting certain criteria (e.g. selecting all objects whose name contains a given string, selecting all objects for which a column value is greater than a specified number).

### Network visualization operations

**Figure 6.**
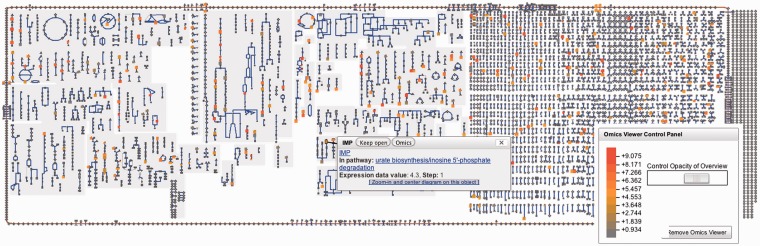
Human metabolic map with metabolites coloured on the basis of a metabolomics data file; mapping of colour values to metabolomics measurements is shown on the right.

Groups can be visualized on three Pathway Tools large-scale diagrams. For example, a metabolite group can be painted onto the Cellular Overview diagram that depicts the metabolic map and transporters. Figure 6 shows a metabolite set highlighted on the human metabolic map. In addition, a gene group can be painted onto the Cellular Overview.

## Implementation Details

Groups are stored and retrieved as serialized Lisp s-expressions generated using an object dumper (18). This utility converts the complex set of interconnected objects that make up a group to or from textual form. Once the textual form is generated, it is stored using one of two possible persistence facilities. The simpler of these stores the textual representation in the file system. This mechanism is suitable for single-user systems, or in cases where security is not an issue.

Multi-user installations of Pathway Tools such as BioCyc.org store the serialized web groups in their associated accounts database. This SQL database holds entries for user accounts, and stored groups are associated with their creator’s account, which permits granting of selective access to particular users. The database schema also has facilities for declaring groups to be publicly readable or writable, and/or for giving read or write access to selected collaborators.

Web infrastructure. Web Groups has a significantly more complex and interactive user interface than did the previously existing Pathway Tools web functionality. Because building complex interactivity on the web is difficult, to speed implementation of Web Groups, we introduced two new layers to the Pathway Tools software infrastructure stack: AllegroServe, a commercially provided Lisp-based web server that replaced the previous web server CWEST (19), and WuWei (20), a toolkit for building complex Ajax applications using Lisp continuations. WuWei runs on top of AllegroServe and provides a number of mechanisms that allow for the construction of dynamic web interfaces. The main WuWei features that enable Web Groups are: (i) The ability to generate persistent Lisp continuations that can be accessed though dynamically generated URLs (21). (ii) The ability to drive Ajax page updates (such as adding a row/column or otherwise updating a page without reloading it) from the back-end Lisp server. (iii) The ability to transparently support session state variables in Lisp.

Performance. The performance of Groups is quite acceptable for interactive use. Most operations complete in less than one second, for example, transforming the group of all 2422 *E. coli* metabolites to the pathways containing them takes less than one second.

## Empirical Usage of Groups at BioCyc.org

### Usage statistics

We analysed the groups existing on the BioCyc.org site using data gathered on 18 February 2013, excluding groups created by SRI internal users (those with @sri.com addresses). Note that our data excludes deleted groups, e.g. if a user created 50 groups and then deleted 40 of them by 18 February 2013, our data would say the user had created 10 groups.

In the 30 days previous to (and including) 18 February 2013, 68 users created or edited groups, giving a sense of the size of the ‘active’ user base. At the time these data were captured, 748 users had at least 1 group, 331 users had exactly 1 group, 359 users had between 2 and 10 groups, 34 users had between 11 and 20 groups and 24 users had more than 20 groups. Single users each had 139, 96, 76, 75 and 58 groups. Most users (597) had groups with only one column; 112 users had groups with up to two columns; 27 users up to 3 columns; and only one user each had groups with up to four and up to five columns. The most prolific Groups user, with 139 groups, created groups of up to four columns.

### Informal survey

To assess the extent to which the BioCyc community finds Groups useful, we invited roughly 1200 users to complete a web-based survey consisting of 18 questions. Of the 96 responses, we selected 43 for further analysis, for those who indicated that they used Groups: ‘Some but not often’, ‘Around half the time’, ‘More than half the time’ or ‘A great deal’.

As described at the outset of this article, one goal of Groups is to help biologists who are not expert in bioinformatics or programming manipulate their data in combination with BioCyc objects, and to thereby make discoveries or conduct analyses that would otherwise require a programmer’s help to accomplish. In all, 77% (30) of those responding to the question of whether Groups achieved this goal reported that it did.

Of those using Groups, 74% (38) reported that they used Groups for exploration; 58% reported using it for analysis; and 32% indicated that they used Groups both for analysis and for exploration. Of the 30 respondents who answered the question about overall usefulness, 97% reported the Groups facility to be somewhat (40%) or very (57%) useful.

Six respondents reported using Groups to create analyses that led either to internal or external publications [e.g. (22)].

#### Survey: most useful features

Those features of Groups that respondents mentioned as being the most useful included its transformation and set operations, its over-representation analysis, its ability to share and download groups for further analysis, the ability to conduct metabolic pathway comparisons and the ability to view properties of different genes ‘all at once’.

Although learning to use Groups was not trivial for some users, others found the online learning resources to be effective. For example, whereas one user reported: ‘The interface is not that intuitive. It took a bit to find out how to upload data’, another reported: ‘I watched the Groups tutorial video on the SRI website, and I was good to go.’ Possibly different sorts of training materials should be offered for different kinds of users.

#### Survey: suggestions for improvement

Respondents reported many areas where Groups could be improved. Most of these were simple suggestions or bug reports that, although well worth implementation or repair, do not merit discussion here. One user requested the ability to create groups that cross organisms. That would permit users to perform what would amount to between-species metabolic or genomic ‘joins’. This would be an interesting computation, and raises the question of what common keys make sense between organisms; such keys would be likely to vary from case to case, but homologous genes at least, or those whose product proteins have the same biochemical function, would make sensible join keys.

## Conclusions

Groups is an implementation of knowledge spreadsheets for Pathway Tools. Groups operates over objects and relationships from Pathway/Genome Databases. The Pathway Tools web-mode implementation of Groups is highly interactive, and provides a variety of operations for creating, modifying, exporting and sharing groups among colleagues. Groups transformations enable users to convert groups from one type of object to another, such as converting a pathway list to a list of all genes or all metabolites within the pathway. Enrichment analysis on Groups detects statistically over-represented sets of entities within a group. At least one group was created at BioCyc.org by 748 users, and 54 users created >10 groups. An informal survey of Groups users suggests that Groups has succeeded in its goal of allowing biologists to perform analyses that previously would have required the assistance of a programmer.

## Software Availability

Groups is available as part of the Pathway Tools software, and is subject to the licensing terms of Pathway Tools: the software is freely available to academic users including source code, and is available for a fee to commercial users. The software is downloadable from (23). The Groups implementation does not function independently of Pathway Tools.
